# Effect of length of time from diagnosis to treatment on colorectal cancer survival: A population-based study

**DOI:** 10.1371/journal.pone.0210465

**Published:** 2019-01-14

**Authors:** Yung-Heng Lee, Pei-Tseng Kung, Yueh-Hsin Wang, Wei-Yin Kuo, Su-Ling Kao, Wen-Chen Tsai

**Affiliations:** 1 Department of Health Services Administration, China Medical University, Taichung, Taiwan, ROC; 2 Department of Public Health, China Medical University, Taiwan, ROC; 3 Department of Orthopedics, Miaoli General Hospital, Miaoli, Taiwan, ROC; 4 Department of Nursing Administration, Jen-Teh Junior College of Medicine, Nursing and Management, Miaoli, Taiwan, ROC; 5 Department of Healthcare Administration, Asia University, Taichung, Taiwan, ROC; 6 Department of Medical Research, China Medical University Hospital, China Medical University, Taichung, Taiwan, R.O.C.; 7 Department of Human Resource, Cishan General Hospital, Kaohsiung, Taiwan, ROC; University of Nebraska Medical Center, UNITED STATES

## Abstract

Evidence is limited regarding the effect of diagnosis-to-treatment interval (DTI) on the survival of colorectal cancer (CRC) patients. In addition, previous studies on treatment delay and CRC survival have largely grouped patients from all stages (I-IV) into one cohort. Our study provides analysis on each stage individually. We conducted a retrospective cohort study with 39,000 newly diagnosed CRC patients obtained from the Taiwan Cancer Registry Database from 2004–2010 to examine the effect of DTIs on overall survival. DTIs were divided into 3 groups: ≤ 30 days (36,115 patients, 90.5% of study patients), 31–150 days (2,533, 6.4%), and ≥ 151 days (1,252, 3.15%). Risk of death was increased for DTI 31–150 days (hazard ratio 1.51; 95% confidence interval 1.43–1.59) and DTI ≥ 151 days (1.64; 1.54–1.76) compared to DTI ≤ 30. This risk was consistent across all cancer stages. Additional factors that increased risk of death include male gender, age >75, Charlson Comorbidity Index ≥7, other catastrophic illnesses, lack of multidisciplinary team involvement, and treatment in a low volume center. From these results, we advise that the DTI for all CRC patients, regardless of cancer staging, should be 30 days or less.

## Introduction

There is an estimated 50,630 deaths from colorectal cancer (CRC) in 2018 in the United States, second only to lung cancer [1]. The Taiwan Cancer Registry report showed that in 2014, CRC was the most frequent cancer in men and second most frequent in women [2]. According to the latest updated data from Taiwan Office of Statistics under the Ministry of Health and Welfare, CRC ranked third among men and women in terms of mortality rate in 2017. In 2007, 19.5 out of every 100,000 people died of CRC, which increased to 24.7 out of 100,000 in 2017. In addition, the mortality rate was higher in men (28.1 per 100,000) than women (21.2 per 100,000) [3].

Delays in diagnosis and treatment can happen any time from the onset of cancer symptoms to start of treatment. Many studies have explored whether this delay (diagnosis-to-treatment interval, DTI) would significantly affect the stage of CRC at diagnosis, prognosis, and overall survival. However, DTIs were often inconsistently defined and measured across studies, leading to inconclusive results. For example, Facione took the time from onset of cancer symptoms to start of treatment and divided it into two intervals: patient delay and provider delay. Patient delay was defined as the period from onset of symptoms to the first medical visit, while provider delay was defined as the period from first visit to start of treatment [4]. Dwivedi et al. divided the same period into three intervals: primary delay, the time from onset of symptoms to the first medical visit; secondary delay, the time from first visit to a confirmed diagnosis; and tertiary delay, the time from a confirmed diagnosis to start of treatment [5].

Treatment delays are postulated to be more likely in countries such as the United States and the United Kingdom due to the wait times inherent to their referral systems [6–8]. Taiwan does not have a mandatory referral system, and once patients apply for a Catastrophic Illness Card, any cancer-related treatment costs are waived. This removes financial barriers and improves medical accessibility. Thus, treatment delays secondary to long wait times or financial difficulties are relatively uncommon. Despite this, there are still patients in Taiwan who refuse to accept treatment, or fail to seek medical attention in a timely manner [9, 10]. No conclusions have been reached regarding the effect of DTI on survival of CRC patients in existing literature [11–17], and no studies examine the survival impact stratified by cancer staging. Thus, the purpose of this study is to determine the effect of DTI on the overall risk of death in patients with CRC and the risk of death for each stage of CRC.

## Materials and methods

### Study design

We designed this study as a retrospective cohort study, utilizing patients newly diagnosed with CRC between 2004 and 2010 as our parent population; these patients were followed to the end of 2012. We obtained the study subjects from the Taiwan Cancer Registry Database, and the contributing variables from the Taiwan’s National Health Insurance Research Database and the administrative data set from the Taiwan Death Registry between 2002 and 2012. The Statistics Center of Ministry of Health and Welfare, Taiwan, provided us with the data sets. Before we used all data, all personal identification information had been deleted by the Ministry of Health and Welfare. All data had been fully anonymized before we accessed them and personal privacy was under protection from using these data. The study was approved by the Institutional Review Board of Cheng Ching Hospital. The IRB number is HP150003.

### Patient selection

We selected patients with Stage I to IV CRC whose ICD-O-3 was C180—C218 as our study subjects. Exclusion criteria were as follows: unknown stage, carcinoma in situ, multiple cancers, having received only palliative treatment for the past year, and missing personal information (such as monthly salary or area of residence) and unknown healthcare provider. The Taiwan Death Registry was used to determine whether a study subject was dead or alive.

### Study variables

The main independent variable in this study is the diagnosis-to-treatment interval (DTI). Control variables include patient profile (gender and age), economic status (monthly income), environmental factor (degree of urbanization), patient’s health condition (catastrophic illnesses except for cancer, Charlson Comorbidity Index [CCI], cancer staging), hospital attributes (hospital level and ownership, service volume), and involvement of a multidisciplinary team (MDT). The dependent variable is overall survival.

We defined DTI as the time interval from a confirmed, pathologic diagnosis of CRC from biopsy to start of treatment (surgery, chemotherapy, or radiotherapy). In this study, we divided DTIs into three categories: ≤ 30 days, 31–150 days, and ≥ 151 days.

Monthly salary was used as a marker of a patient’s economic status. We divided income into 7 levels: low-income households, <NT$17,800, NT$17,281–22,800, NT$22,801–28,000, NT$28,801–36,300, NT$36,301–45,800, and >NT$45,801. For environmental factors, we assigned each patient’s place of residence a score indicating degree of urbanization, with Level 1 as the highest degree of urbanization and Level 7 the lowest [18]. Our assessment of each patient’s health status included the presence of catastrophic illnesses except for cancer, CCI, and cancer staging. We defined ‘catastrophic illness’ according to the National Health Insurance Administration definition, which includes 30 diseases such as malignant neoplasm, hemophilia, hemolytic anemias, chronic renal failure, stroke, insulin dependent diabetes mellitus, systemic sclerosis, and systemic lupus erythematosus. For patients with a qualifying health condition, expenses related to medical treatment are waived. In addition, we measured a patient’s degree of burden from comorbidities with the Deyo-modified Charlson Comorbidity Index. The CCI was shown to be significantly correlated with factors such as postoperative complications, death rate, admission days and cost of hospitalization and is divided into three levels: ≤ 3, 4–6, and ≥ 7 [19]. For cancer staging, we used the American Joint Committee on Cancer (AJCC) sixth edition staging guidelines for CRC [20].

We defined the primary healthcare facility as a patient’s main treatment center, and divided them into four tiers: major medical center, regional hospital, local hospital, and other healthcare facilities. We also determined whether the hospital ownership was public or non-public, and evaluated their annual service volume according to quartiles: low, <25%; mid, 25–75%; and high, >75%. With regards to the MDT, we defined this as a multidisciplinary group including surgeons, medical oncologists, radiation oncologists, and members of allied health such as psychologists, physical therapists, dieticians, pharmacists, cancer nurses, cancer case managers, hospices, and discharge planning staff members who all work together to provide comprehensive cancer care [21].

### Statistical analysis

In this study, we utilized descriptive statistics to analyze different DTIs and the distribution of control variables (including patient profile, economic status, environmental factor, health condition of the patient, hospital attributes, and MDT involvement). We used the log-rank test for inferential statistical analysis to test for statistically significant differences (P<0.05) between different DTIs and the respective control variables on the survival of CRC patients.

The Cox proportional hazards model was used to analyze the effect of different DTIs on the risk of death of patients with CRC as a whole, and that of patients with CRC in respective stages after controlling for related variables (including patient profile, economic status, environmental factor, health conditions, hospital attributes, and MDT involvement). We used months as the unit of measurement for survival. When an observed patient died before the end of 2012, we defined this as an event; if they were alive by the end of 2012 or had withdrawn from National Health Insurance coverage without subsequent data available for observation, we defined this as censored.

We constructed the survival curve of different DTIs versus patients with CRC as a whole and patients with CRC in different stages after controlling for confounding variables as listed above.

Finally, we designated our study population for each CRC stage as the derivation cohort, and randomly selected 10% from each stage as our validation cohort. Bivariate correlation analyses were performed between the derivation cohort and the validation cohort for each cancer stage. We draw the receiver operating characteristic (ROC) curves and calculated the area under curves (AUCs) for the validation cohort by different cancer stage.

In this study, all statistical analyses were performed using the SAS software, version 9.3 (SAS Institute Inc., Cary, NC). All tests were two-tailed, with α = 0.05 as the confidence interval.

## Results

### Enrollment and patient characteristics

In the Taiwan Cancer Registry Database, there were a total of 64,241 patients with newly confirmed diagnosis of CRC between 2004 and 2010. After excluding those with unknown disease stage (18,612), carcinoma in situ (3,070), multiple cancers (973), palliative treatment in the past year (360), and missing personal information or unknown healthcare provider (1,326), a total of 39,900 patients were obtained as the study population, with an average age of 65.1±13.4 years. These patients were followed until 2012; a total of 22,614 patients (56.7%) survived and 17,286 (43.3%) died (Table 1).

**Table 1:** Descriptive statistics of colorectal cancer patients based on survival status.

Variables	Total		Alive		Death		P value
	N	%	N	%	N	%	
**Total number**	39,900	100.00	22,614	56.68	17,286	43.32	-
**Interval from cancer diagnosis to treatment**							<0.001
≤ 30 days	36,115	90.51	21,342	59.09	14,773	40.91	
31~150 days	2,533	6.35	980	38.69	1,553	61.31	
≥ 151 days	1,252	3.14	292	23.32	960	76.68	
**Gender**							<0.001
Female	17,030	42.68	10,057	59.05	6,973	40.95	
Male	22,870	57.32	12,557	54.91	10,313	45.09	
**Age**							<0.001
≤ 44	2,883	7.23	1,684	58.41	1,199	41.59	
45~54	6,031	15.12	3,812	63.21	2,219	36.79	
55~64	9,088	22.78	5,928	65.23	3,160	34.77	
65~74	10,720	26.87	6,346	59.20	4,374	40.80	
≥ 75	11,178	28.02	4,844	43.34	6,334	56.66	
**Mean age**	65.13	13.44	63.48	12.71	67.28	14.05	<0.001
**Monthly salary**							<0.001
Low-income	338	0.85	136	40.24	202	59.76	
≤ 17280	1,521	3.81	852	56.02	669	43.98	
17281~22800	19,942	49.98	10,820	54.26	9,122	45.74	
22801~28800	7,581	19.00	4,261	56.21	3,320	43.79	
28801~36300	2,709	6.79	1,694	62.53	1,015	37.47	
36301~45800	3,468	8.69	2,167	62.49	1,301	37.51	
≥ 45801	4,341	10.88	2,684	61.83	1,657	38.17	
**Urbanization level**							<0.001
Level 1	11,265	28.23	6,441	57.18	4,824	42.82	
Level 2	11,709	29.35	6,819	58.24	4,890	41.76	
Level 3	6,176	15.48	3,471	56.20	2,705	43.80	
Level 4	5,863	14.69	3,212	54.78	2,651	45.22	
Level 5	1,228	3.08	701	57.08	527	42.92	
Level 6	1,780	4.46	911	51.18	869	48.82	
Level 7	1,879	4.71	1,059	56.36	820	43.64	
**CCI score**							<0.001
≤ 3	25,962	65.07	17,646	67.97	8,316	32.03	
4~6	6,208	15.56	2,829	45.57	3,379	54.43	
≥ 7	7,730	19.37	2,139	27.67	5,591	72.33	
**Catastrophic illness**							<0.001
No	38,459	96.39	22,039	57.31	16,420	42.69	
Yes	1,441	3.61	575	39.90	866	60.10	
**Cancer stage**							<0.001
Stage I	6,448	16.16	5,286	81.98	1,162	18.02	
Stage II	9,555	23.95	6,772	70.87	2,783	29.13	
Stage III	13,285	33.30	8,904	67.02	4,381	32.98	
Stage IV	10,612	26.60	1,652	15.57	8,960	84.43	
**Joint MDT care**							<0.001
No	35,362	88.63	19,768	55.90	15,594	44.10	
Yes	4,538	11.37	2,846	62.71	1,692	37.29	
**Hospital level**							<0.001
Major medical center	26,475	66.35	15,163	57.27	11,312	42.73	
Regional hospital	12,663	31.74	7,186	56.75	5,477	43.25	
District hospital	642	1.61	206	32.09	436	67.91	
Others	120	0.30	59	49.17	61	50.83	
**Hospital ownership**							<0.001
Public	11,930	29.90	6,641	55.67	5,289	44.33	
Private	27,970	70.10	15,973	57.11	11,997	42.89	
**Hospital service volume**							<0.001
Low	10,018	25.11	4,954	49.45	5,064	50.55	
Middle	19,823	49.68	11,268	56.84	8,555	43.16	
High	10,059	25.21	6,392	63.55	3,667	36.45	

The DTIs for CRC in this study were divided into three groups: ≤ 30 days, 31–150 days, and ≥ 151 days. 36,115 study subjects (90.5%) received treatment within 30 days of a confirmed diagnosis; 2533 (6.4%) did so between 31–150 days; and 1,252 (3.1%) did not receive treatment until more than 151 days after diagnosis. In addition, patient survival was significantly impacted (p < 0.05) by variables such as gender, age, monthly income, degree of urbanization in area of residence, Charlson Comorbidity Index (CCI), presence of catastrophic illnesses except for cancer, cancer staging, involvement of a multidisciplinary team (MDT), and hospital factors (hospital level and ownership, responsibilities, and service volume).

#### Factors associated with overall survival in colorectal cancer patients

Table 2 shows the risk of death in terms of hazard ratios (HR) with different DTIs as determined by the Cox proportional hazards model, controlling for all other variables. Our results show that patients with longer DTIs had greater risk of death. With patients receiving treatment within 30 days of a confirmed diagnosis as the reference group, the risk of death associated with starting treatment between 31–150 days and after 151 days of a confirmed CRC diagnosis were 1.51 times (95% confidence interval: 1.43–1.59) and 1.64 times (95% CI: 1.54–1.76) greater, respectively. Examining other variables, the risk of death was 1.1 times (95% CI: 1.08–1.15) greater in men than in women. The risk of death among patients aged 45–64 was lower than those younger than 44 (HR = 0.90). For patients older than 75, however, the risk of death was 1.77 times (95% CI: 1.66–1.89) greater than those aged 44 or below. In addition, a higher salary was associated with lower risk of death; for example, patients with monthly income greater than NTD 45801 had a risk of death only 0.64 times that of patients with lower monthly income. As well, the higher the CCI score, the higher the risk of death; a CCI score of ≥7 was associated with a risk of death 1.68 times (95% CI: 1.62–1.74) higher than a CCI score of ≤3. Similarly, presence of catastrophic illnesses except for cancer was related to a greater risk of death (HR 1.7, 95% CI: 1.59–1.83). As expected, a higher cancer stage was associated with greater risk of death. The risk of death from Stage IV disease was 8.86 times (95% CI: 8.32–9.45) that of Stage I disease. Lastly, lower risk of death was seen with involvement of an MDT (HR: 0.94; 95% CI: 0.89–0.99) and high hospital service volume (HR: 071; 95% CI: 0.67–0.74).

**Table 2:** Factors associated with overall survival in colorectal cancer patients.

Variables	Unadjusted		Adjusted			
	HR	P value	HR	95% CI		P value
**Interval from cancer diagnosis to treatment**						
≤ 30 days (ref.)						
31~150 days	**1.93**	<0.001	**1.51**	1.43	1.59	<0.001
≥ 151 days	**3.08**	<0.001	**1.64**	1.54	1.76	<0.001
**Gender**						
Female (ref.)						
Male	**1.14**	<0.001	**1.11**	1.08	1.15	<0.001
**Age**						
≤ 44 (ref.)						
45~54	**0.86**	<0.001	**0.90**	0.84	0.96	0.002
55~64	**0.81**	<0.001	**0.90**	0.84	0.96	0.002
65~74	**0.98**	0.585	**1.09**	1.02	1.16	0.009
≥ 75	**1.60**	<0.001	**1.77**	1.66	1.89	<0.001
**Monthly salary**						
Low-income (ref.)						
≤ 17280	**0.60**	<0.001	**0.78**	0.66	0.91	0.002
17281~22800	**0.64**	<0.001	**0.75**	0.65	0.86	<0.001
22801~28800	**0.62**	<0.001	**0.74**	0.64	0.85	<0.001
28801~36300	**0.52**	<0.001	**0.72**	0.61	0.83	<0.001
36301~45800	**0.50**	<0.001	**0.69**	0.59	0.80	<0.001
≥ 45801	**0.50**	<0.001	**0.64**	0.56	0.74	<0.001
**Urbanization level**						
Level 1 (ref.)						
Level 2	**0.97**	0.118	**0.96**	0.93	1.00	0.077
Level 3	**1.04**	0.102	**1.03**	0.98	1.08	0.294
Level 4	**1.07**	0.003	**1.01**	0.96	1.06	0.843
Level 5	**1.02**	0.746	**0.94**	0.86	1.04	0.223
Level 6	**1.20**	<0.001	**1.02**	0.95	1.10	0.635
Level 7	**1.06**	0.128	**1.04**	0.97	1.12	0.299
**CCI score**						
≤ 3 (ref.)						
4~6	**2.05**	<0.001	**1.42**	1.37	1.48	<0.001
≥ 7	**3.49**	<0.001	**1.68**	1.62	1.74	<0.001
**Catastrophic illness**						
No (ref.)						
Yes	**1.82**	<0.001	**1.70**	1.59	1.83	<0.001
**Cancer stage**						
Stage I (ref.)						
Stage II	**1.65**	<0.001	**1.58**	1.48	1.69	<0.001
Stage III	**1.98**	<0.001	**1.94**	1.82	2.08	<0.001
Stage IV	**10.48**	<0.001	**8.86**	8.32	9.45	<0.001
**Joint MDT care**						
No (ref.)						
Yes	**0.83**	<0.001	**0.94**	0.89	0.99	0.017
**Hospital level**						
Major medical center (ref.)						
Regional hospital	**1.12**	<0.001	**0.96**	0.92	1.00	0.030
District hospital	**2.25**	<0.001	**1.40**	1.26	1.55	<0.001
Others	**1.29**	0.045	**0.96**	0.74	1.24	0.743
**Hospital ownership**						
Public (ref.)						
Private	**0.94**	<0.001	**0.99**	0.96	1.02	0.490
**Hospital services volume**						
Low (ref.)						
Middle	**0.80**	<0.001	**0.85**	0.82	0.89	<0.001
High	**0.69**	<0.001	**0.71**	0.67	0.74	<0.001

**Fig 1:**
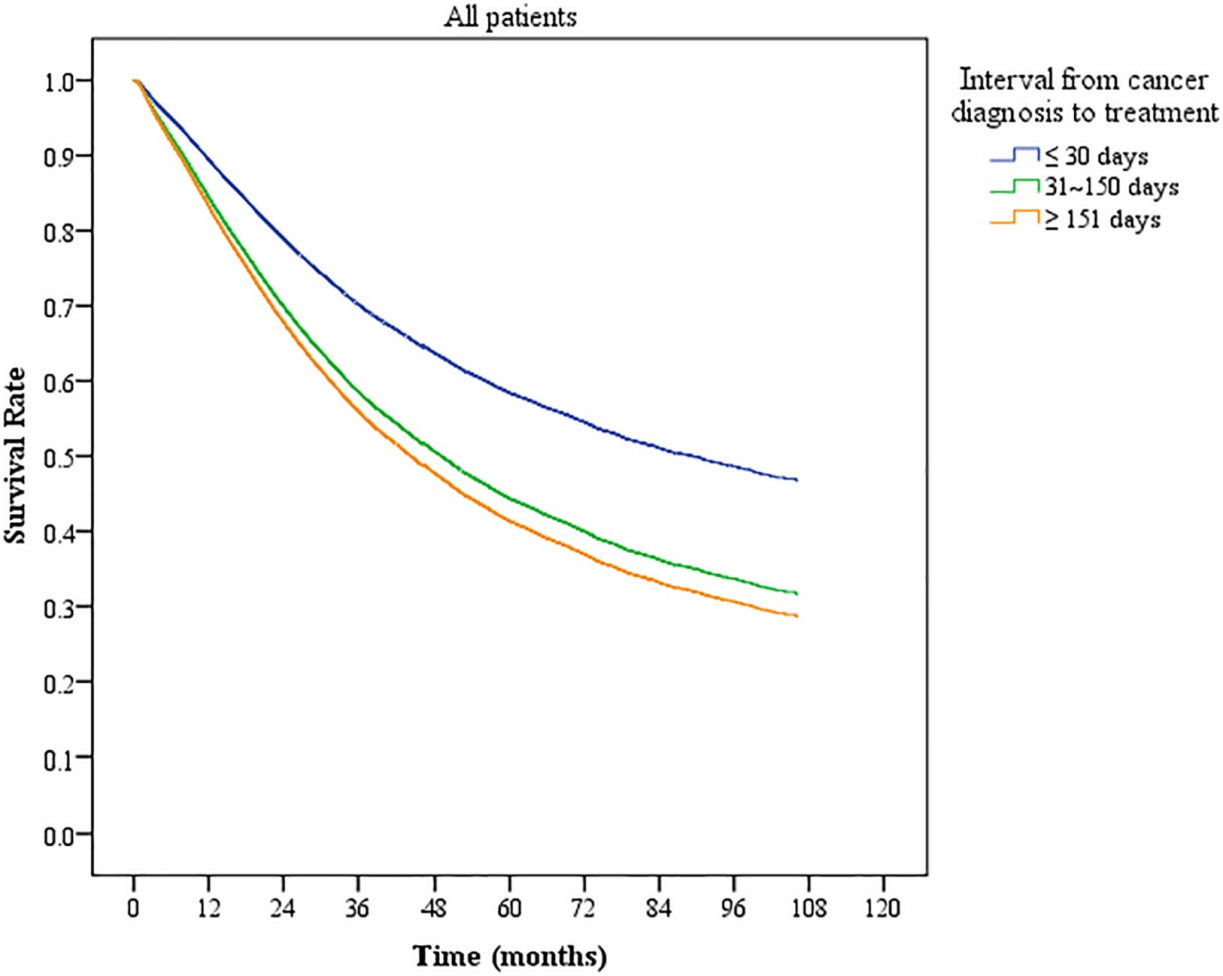
Overall Cox proportional survival curves of colorectal cancer patients stratified by different diagnosis-to-treatment intervals.

The Fig 1 shows the risk of death with different DTIs as determined by the Cox proportional hazards model, controlling for all other variables. Our results show that patients with longer DTIs had greater risk of death. Patients receiving treatment within 30 days of a confirmed diagnosis had the highest survival rate.

#### Overall survival of different DTIs based on cancer stage

**Fig 2:**
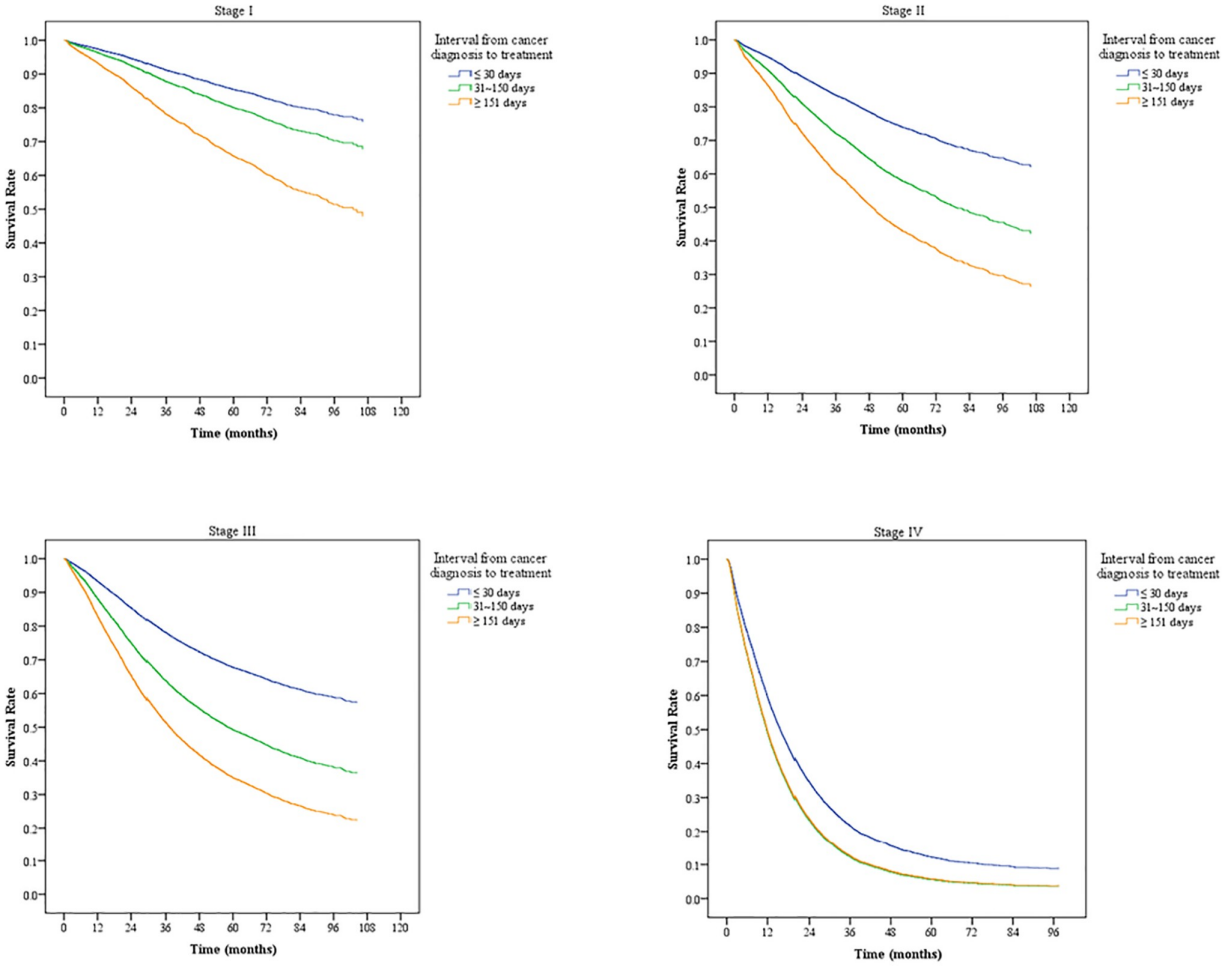
Overall survival curves of different diagnosis-to-treatment intervals based on cancer stage.

After controlling for each variable, we found that longer DTIs increased the risk of death in Stages I, II, and III through regression analysis (Fig 2).

In patients with Stage I disease, with DTI ≤ 30 days as the reference group, the risk of death was 1.41 times greater at DTI 31–150 days (95% CI: 1.25–1.47), and 2.66 times greater at DTI ≥ 151 days (95% CI: 2.09–3.40). In patients with Stage IV disease, the risk of death at DTI 31–150 days was the same as DTI ≥ 151 days (1.37, 95% CI: 1.28–1.47, and 1.36, 95% CI: 1.25–1.47, respectively) (Table 3).

**Table 3:** Overall survival of different time intervals from diagnosis to treatment based on tumor stage.

Variables	Stage I				Stage II				Stage III				Stage IV			
	HR	95% CI		P value	HR	95% CI		P value	HR	95% CI		P value	HR	95% CI		P value
**Interval from cancer diagnosis to treatment**																
≤ 30 days (ref.)	**1.00**				**1.00**				**1.00**				**1.00**			
31~150 days	**1.41**	1.14	1.74	0.001	**1.81**	1.56	2.11	<0.001	**1.82**	1.61	2.06	<0.001	**1.37**	1.28	1.47	<0.001
≥ 151 days	**2.66**	2.09	3.40	<0.001	**2.80**	2.28	3.43	<0.001	**2.70**	2.27	3.20	<0.001	**1.36**	1.25	1.47	<0.001

Note: Variables such as gender, age, monthly salary, urbanization level, CCI, catastrophic illness, joint MDT care, hospital level, hospital ownership and hospital services volume are controlled.

#### Analysis of the validation cohort based on cancer stage

**Fig 3:**
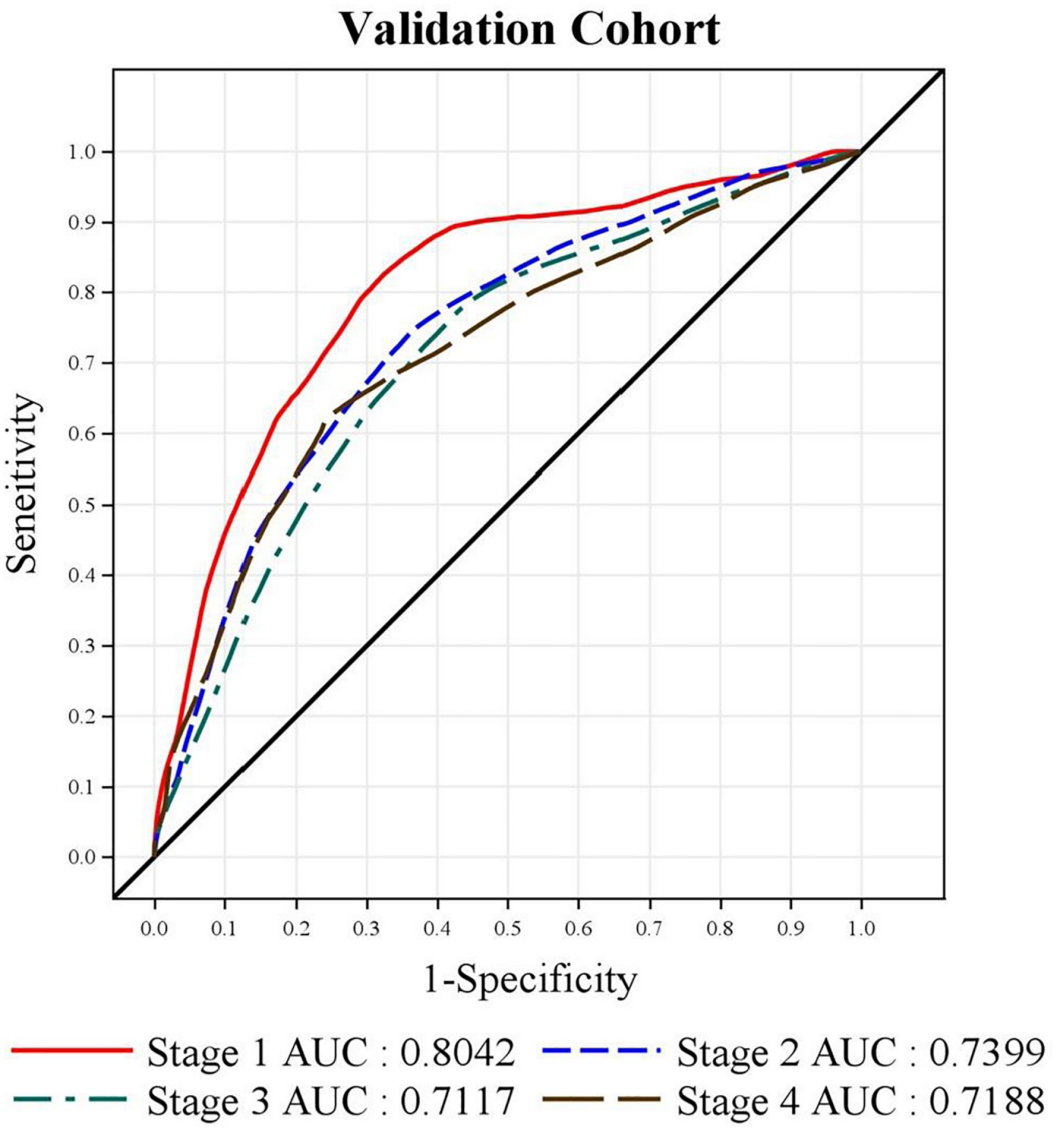
ROC curve (receiver operating characteristic curve) and area under curves (AUCs) of the validation cohort by different cancer stages.

We performed bivariate correlation analyses between the derivation cohort and the validation cohort for each cancer stage. Both cohorts were found to be similar in distribution across all cancer stages (p value > 0.05) (S1 and S2 Tables). We constructed receiver operating characteristic (ROC) curves and calculated the area under curves (AUCs) for the validation cohort for each tumor stages. The AUC for Stage I to IV were 0.80, 0.74, 0.71, and 0.72, respectively (Fig 3), which were over 0.70.

**Fig 4:**
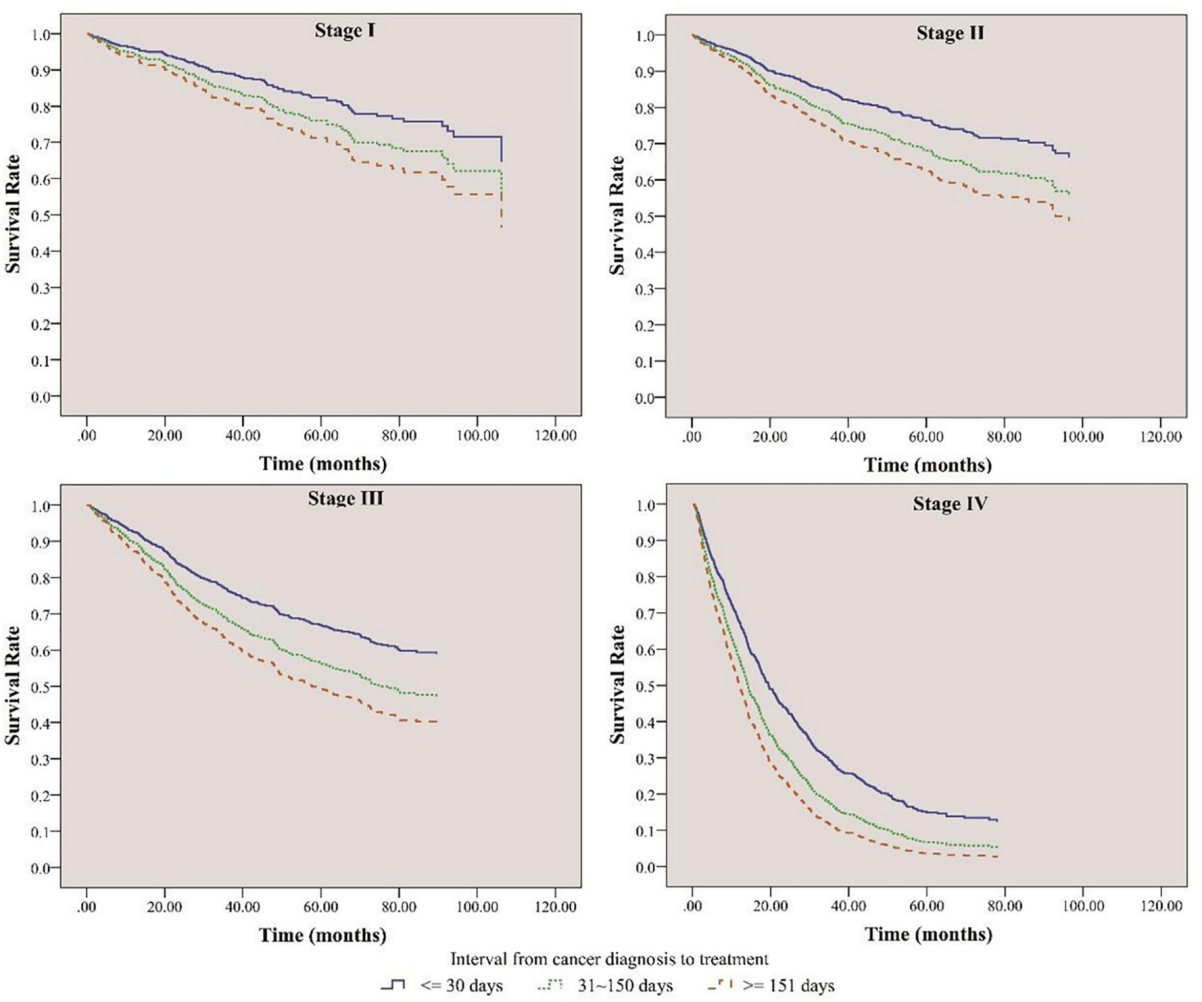
Overall survival curves of different diagnosis-to-treatment intervals for the validation cohort by cancer stages.

We also used the validation cohort to plot the survival curves of different diagnosis-to-treatment intervals by cancer stages (Fig 4). We found that longer DTIs increased the risk of death in the validation cohort (Fig 4), which was similar to those in the derivation cohort (Fig 2).

## Discussion

There have been multiple studies exploring the impact of delay in treatment on risk of death in CRC; however, no conclusions have been definitively reached. In many publications, treatment delay has not been found to decrease survival of CRC patients [11, 14–17]. However, those studies did not include a sufficiently large sample size, and currently there are no studies that investigate the effect of DTIs on CRC survival for each stage of disease. In this study, we accessed the nationwide database to obtain a large enough sample size to conduct robust statistical analysis. A total of 39,900 patients with CRC were analyzed and results showed that longer DTI was associated with higher risk of death. The risk of death with treatment between 31–150 days and after 151 days were 1.51 times and 1.64 times higher, respectively, than those who received treatment within 30 days following confirmed diagnosis of cancer. Decreased survival with longer DTIs was again seen when stratified according to stage of disease, confirming that the risk of death was lowest for any patient if they receive treatment within 30 days of confirmed diagnosis. This finding is in keeping with the NHS Cancer Plan executed by the United Kingdom in 2000, which proposed the goal of reducing wait times from diagnosis to treatment for all cancers to one month or less by 2005 [22]. In addition, timely treatment provided to patients after a confirmed diagnosis helps to reduce their psychological stress and anxiety, and improve quality of life [23]. Therefore, we recommend that the DTI for all CRC patients, regardless of disease staging, should be 30 days.

Existing literature defines ‘treatment delay’ as DTI longer than 30 days [24, 25]. Irene et al. found that treatment delay occurred in 65.5% of CRC patients in Spain [24]. Abu-Helalah et al. found that treatment delay occurred in 32.6% of CRC patients in Jordan [25]. Studies in the USA have also shown that the average waiting time has grown longer and longer in recent years [26]. As shown in our study, 90.5% of Taiwanese CRC patients started treatment within 30 days of a confirmed diagnosis, which meant treatment delay only occurred in 9.5% of patients. Explanations for this include the existence of National Health Insurance in Taiwan, and the ability of cancer patients to apply for a Catastrophic Illness Card, which waives any medical expenses involved in the cancer treatment. In addition, no mandatory referral system exists in Taiwan, thereby minimizing delays; and tertiary care centers in Taiwan handle a high density of cancer cases, which translate to greatly improve medical accessibility for patients. As a result, patients in Taiwan are less likely to experience delays in their cancer treatment.

A large European study (EUROCARE) found that the risk of death was higher in men than women with CRC (HR = 1.05–1.08) [27]. In the US, population data from 2009–2013 showed that the rate of death from CRC was 1.4 times higher in men than women (95% CI: 1.42–1.44) [1]. The results obtained in our study are similar, with men having a greater risk of death (HR = 1.11), and match the findings in our previous study [21]. In terms of the effect of age on the risk of death, however, the results of this study differed from the EUROCARE study [27]. EUROCARE determined that as age increased, the risk of death increased as well (HR = 1.20–2.63). In our study, the lowest risk of death was not found within the youngest age group (age<45) but within those aged 45–64 (HR = 0.90). This finding is similar to that of the SEER (Surveillance, Epidemiology, and End Results) registry study between 2001 and 2010 in the US [28]. The SEER registry study showed that CRC ranked first in prevalence and death rate among all cancers in Americans younger than 50, and the rates are rising. High death rates in young patients with CRC are thought to be due to longer treatment delays and higher staging at time of diagnosis. Similar studies in Taiwan have also shown a higher stage and worse prognosis among young patients with CRC [21, 29, 30].

Studies have indicated that patients with higher socioeconomic status (SES) have better health awareness and are more involved in cancer screening. As a result, their stage of cancer at time of diagnosis are often less advanced [31]. Compared to those with high SES, patients with low SES are less likely to receive cancer screening and adjuvant treatments such as radiotherapy or chemotherapy, and have greater risk of death (HR = 1.3–1.8) [32]. In addition, they are at higher risk of death from any cause compared to those with high SES [33]. Our current study also showed similar findings; with low income earners as the reference group, the higher the monthly salary, the lower the risk of death. CRC patients with a monthly salary greater than NTD 45801 have a risk of death 0.64 times that of those with lower income. In addition, studies have found correlation between monthly salary and degree of co-morbidities. The risk that cancer patients with low SES have at least one co-morbidity is 50% higher than patients with high SES [34]. Co-morbidities affect the stage of cancer at diagnosis, the treatment options available, and the overall prognosis, leading to an increased risk of death [35, 36]. Results of this study showed that the risk of death among patients with higher CCI scores was also greater. A CCI score of ≥ 7 conferred a risk of death 1.68 times higher than a CCI score of ≤ 3.

Studies have shown that patients with CRC who receive treatment or surgery at a large volume center may have better overall survival [21, 37, 38]. Large volume centers are thought to be better trained and equipped to provide higher quality care, including better surgical resections, more comprehensive multidisciplinary team involvement, more frequent follow up and surveillance, and fewer treatment complications [37, 38]. A Taiwanese study compared outcomes between low and high service volume hospitals and found lower rates of death in high volume centers for patients with CRC (HR = 0.55) [21]. Similarly, our study showed the risk of death among patients treated in high service volume facilities was indeed significantly lower (HR = 0.67).

In 2003, the Taiwan Health Promotion Administration, Ministry of Health and Welfare introduced a plan called “Cancer Centers for a Great Improvement in the Quality of Cancer Care” which promoted the MDT framework for care of cancer patients. After patients with CRC join the MDT cancer care network, the medical team will consist of not only the attending physician but also radiologists, psychologists, physical therapists, dieticians, pharmacists, cancer nurses, cancer case managers, hospice care staff and discharge planning staff. The MDT provides integrated patient-centered care resulting in more comprehensive treatment plans and better quality of care. The benefit of MDT policies on the prognosis of patients with CRC was studied in literature and results conclusively show significantly higher survival in CRC patients treated with a MDT [39–41]. A Taiwanese study with 25,766 CRC patients found that the risk of death among CRC patients with MDT involvement was lower than patients without MDT care (HR = 0.91) [21]. Our study revealed similar findings, with MDT involvement decreasing the risk of death (HR = 0.94).

Finally, we found that the AUC for Stages I to IV were 0.80, 0.74, 0.71, and 0.72, respectively. An AUC above 0.8 indicates excellent predictive power, whereas AUCs between 0.7–0.8 are considered moderate and 0.6–0.7 are considered poor. Through the above validation cohort analyses, we conclude that our study results show acceptable discrimination, which further strengthens the findings and conclusion of our study.

Limitations of this study stem from the fact that data were obtained from a secondary database. Therefore, potentially confounding factors such as presenting symptoms and signs, family history, and lifestyle factors including diet and physical activity were unavailable, and could not be included in the study analysis. We selected 64,241 patients with Stage I to IV CRC as our study subjects, but 24,341 patients with unknown stage, carcinoma in situ, multiple cancers, having received only palliative treatment for the past year, and missing personal information and unknown healthcare provider were excluded. Although the dropout rate reaches 37.9%, the sufficiently large sample size and robust analysis lends confidence that the final results are not significantly affected.

In conclusion, this study found that a longer interval from a confirmed diagnosis of CRC to start of treatment was associated with a significantly higher risk of death. This result was consistent across all cancer stages. As our data show, the sooner patients with CRC receive treatment, the better the survival rate. Contributing factors include the patient’s age and gender, their economic status and overall health condition, as well as attributes of the treating hospital and involvement of a multidisciplinary team. It is advised through this study that the DTI for CRC patients should be within 30 days. In addition, as the factors underlying treatment delay were not included in this study, we believe future studies examining this area may be beneficial.

## Supporting information

S1 Table Bivariate correlation analysis between the derivation cohort and the validation cohort for cancer stage 1 & 2. (DOCX)

S2 Table Bivariate correlation analysis between the derivation cohort and the validation cohort for cancer stage 3 & 4. (DOCX)

## Abbreviations

AJCC: American Joint Committee on Cancer
CCI: Charlson Comorbidity Index
CI: confidence interval
CRC: colorectal cancer
DTI: diagnosis-to-treatment interval
HR: Hazard ratio
MDT: multidisciplinary team
SES: socioeconomic status
