# Supplementary material for: Effect of length of time from diagnosis to treatment on colorectal cancer survival: A population-based study
Source: PLoS One. 2019 Jan 14;14(1):e0210465. doi: 10.1371/journal.pone.0210465 (PMC6331126; doi:10.1371/journal.pone.0210465)
Supplement: S1 Table — (DOCX) [file pone.0210465.s001.docx]

| **S1 Table. Bivariate correlation analysis between the derivation cohort and the validation cohort for cancer stage 1 & 2** | | | | | | | | | | | |
| --- | --- | --- | --- | --- | --- | --- | --- | --- | --- | --- | --- |
|  | |  | **Stage 1** |  |  |  | **Stage 2** | |  |  |  |
| **Variables** | | **Derivation Cohort** | | **Validation Cohort** | | **P value** | **Derivation Cohort** | | **Validation Cohort** | | **P value** |
|  |  | **N** | **%** | **N** | **%** |  | **N** | **%** | **N** | **%** |  |
| **Total number** | | 6,448 | 100.00 | 645 | 100.00 | - | 9,555 | 100.00 | 956 | 100.00 | - |
| **Interval from cancer**  **diagnosis to treatment** | |  |  |  |  | 0.762 |  |  |  |  | 0.278 |
|  | ≤ 30 days | 5,867 | 90.99 | 591 | 91.63 |  | 8,964 | 93.81 | 908 | 94.98 |  |
|  | 31~150 days | 411 | 6.37 | 40 | 6.20 |  | 425 | 4.45 | 37 | 3.87 |  |
|  | ≥ 151 days | 170 | 2.64 | 14 | 2.17 |  | 166 | 1.74 | 11 | 1.15 |  |
| **Gender** | |  |  |  |  | 0.032 |  |  |  |  | 0.326 |
|  | Female | 2,777 | 43.07 | 249 | 38.60 |  | 4,035 | 42.23 | 420 | 43.93 |  |
|  | Male | 3,671 | 56.93 | 396 | 61.40 |  | 5,520 | 57.77 | 536 | 56.07 |  |
| **Age** | |  |  |  |  | 0.976 |  |  |  |  | 0.753 |
|  | ≤ 44 | 352 | 5.46 | 36 | 5.58 |  | 560 | 5.86 | 53 | 5.54 |  |
|  | 45~54 | 939 | 14.56 | 88 | 13.64 |  | 1,258 | 13.17 | 129 | 13.49 |  |
|  | 55~64 | 1,582 | 24.53 | 157 | 24.34 |  | 2,056 | 21.52 | 200 | 20.92 |  |
|  | 65~74 | 1,913 | 29.67 | 194 | 30.08 |  | 2,645 | 27.68 | 252 | 26.36 |  |
|  | ≥ 75 | 1,662 | 25.78 | 170 | 26.36 |  | 3,036 | 31.77 | 322 | 33.68 |  |
| **Mean age** | | 65.28 | 12.52 | 65.36 | 12.41 | 0.871 | 66.69 | 13.20 | 66.96 | 13.44 | 0.558 |
| **Monthly salary** | |  |  |  |  | 0.298 |  |  |  |  | 0.845 |
|  | Low-income | 41 | 0.64 | 31 | 4.81 |  | 74 | 0.77 | 8 | 0.84 |  |
|  | ≤ 17280 | 218 | 3.38 | 0 | 0.00 |  | 362 | 3.79 | 40 | 4.18 |  |
|  | 17281~22800 | 3,236 | 50.19 | 315 | 48.84 |  | 4,908 | 51.37 | 481 | 50.31 |  |
|  | 22801~28800 | 1,105 | 17.14 | 100 | 15.50 |  | 1,833 | 19.18 | 181 | 18.93 |  |
|  | 28801~36300 | 460 | 7.13 | 43 | 6.67 |  | 588 | 6.15 | 55 | 5.75 |  |
|  | 36301~45800 | 611 | 9.48 | 58 | 8.99 |  | 773 | 8.09 | 90 | 9.41 |  |
|  | ≥ 45801 | 777 | 12.05 | 98 | 15.19 |  | 1,017 | 10.64 | 101 | 10.56 |  |
| **Urbanization level** | |  |  |  |  | 0.732 |  |  |  |  | 0.197 |
|  | Level 1 | 1,734 | 26.89 | 179 | 27.75 |  | 2,595 | 27.16 | 261 | 27.30 |  |
|  | Level 2 | 1,943 | 30.13 | 205 | 31.78 |  | 2,781 | 29.11 | 272 | 28.45 |  |
|  | Level 3 | 946 | 14.67 | 94 | 14.57 |  | 1,515 | 15.86 | 167 | 17.47 |  |
|  | Level 4 | 990 | 15.35 | 87 | 13.49 |  | 1,448 | 15.15 | 128 | 13.39 |  |
|  | Level 5 | 239 | 3.71 | 25 | 3.88 |  | 285 | 2.98 | 20 | 2.09 |  |
|  | Level 6 | 278 | 4.31 | 30 | 4.65 |  | 487 | 5.10 | 52 | 5.44 |  |
|  | Level 7 | 318 | 4.93 | 25 | 3.88 |  | 444 | 4.65 | 56 | 5.86 |  |
| **CCI score** | |  |  |  |  | 0.397 |  |  |  |  | 0.851 |
|  | ≤ 3 | 5,281 | 81.90 | 542 | 84.03 |  | 7,260 | 75.98 | 720 | 75.31 |  |
|  | 4~6 | 827 | 12.83 | 74 | 11.47 |  | 1,348 | 14.11 | 136 | 14.23 |  |
|  | ≥ 7 | 340 | 5.27 | 29 | 4.50 |  | 947 | 9.91 | 100 | 10.46 |  |
| **Catastrophic illness** | |  |  |  |  | 1.000 |  |  |  |  | 0.197 |
|  | No | 6,170 | 95.69 | 617 | 95.66 |  | 9,224 | 96.54 | 931 | 97.38 |  |
|  | Yes | 278 | 4.31 | 28 | 4.34 |  | 331 | 3.46 | 25 | 2.62 |  |
| **Joint MDT care** | |  |  |  |  | 0.230 |  |  |  |  | 0.877 |
|  | No | 5,697 | 88.35 | 559 | 86.67 |  | 8,606 | 90.07 | 859 | 89.85 |  |
|  | Yes | 751 | 11.65 | 86 | 13.33 |  | 949 | 9.93 | 97 | 10.15 |  |
| **Hospital level** | |  |  |  |  | 0.796 |  |  |  |  | 0.953 |
|  | Major medical center | 4,054 | 62.87 | 417 | 64.65 |  | 6,596 | 69.03 | 661 | 69.14 |  |
|  | Regional hospital | 2,275 | 35.28 | 215 | 33.33 |  | 2,765 | 28.94 | 278 | 29.08 |  |
|  | District hospital | 100 | 1.55 | 13 | 2.02 |  | 155 | 1.62 | 14 | 1.46 |  |
|  | Others | 19 | 0.29 |  | 0.00 |  | 39 | 0.41 | 3 | 0.31 |  |
| **Hospital ownership** | |  |  |  |  | 0.708 |  |  |  |  | 0.042 |
|  | Public | 1,719 | 26.66 | 167 | 25.89 |  | 2,986 | 31.25 | 330 | 34.52 |  |
|  | Private | 4,729 | 73.34 | 478 | 74.11 |  | 6,569 | 68.75 | 626 | 65.48 |  |
| **Hospital service volume** | |  |  |  |  | 0.972 |  |  |  |  | 0.505 |
|  | Low | 1,697 | 26.32 | 169 | 26.20 |  | 2,545 | 26.64 | 246 | 25.73 |  |
|  | Middle | 3,119 | 48.37 | 315 | 48.84 |  | 4,769 | 49.91 | 496 | 51.88 |  |
|  | High | 1,632 | 25.31 | 161 | 24.96 |  | 2,241 | 23.45 | 214 | 22.38 |  |
