# Supplementary material for: Effect of length of time from diagnosis to treatment on colorectal cancer survival: A population-based study
Source: PLoS One. 2019 Jan 14;14(1):e0210465. doi: 10.1371/journal.pone.0210465 (PMC6331126; doi:10.1371/journal.pone.0210465)
Supplement: S2 Table — (DOCX) [file pone.0210465.s002.docx]

| **S2 Table. Bivariate correlation analysis between the derivation cohort and the validation cohort for cancer stage 3 & 4** | | | | | | | | | | | |
| --- | --- | --- | --- | --- | --- | --- | --- | --- | --- | --- | --- |
|  | |  | **Stage 3** |  |  |  | **Stage 4** | |  |  |  |
| **Variables** | | **Derivation Cohort** | | **Validation Cohort** | | **P value** | **Derivation Cohort** | | **Validation Cohort** | | **P value** |
|  |  | **N** | **%** | **N** | **%** |  | **N** | **%** | **N** | **%** |  |
| **Total number** | | 13,285 | 100.00 | 1,329 | 100.00 | - | 10,612 | 100.00 | 1,062 | 100.00 | - |
| **Interval from cancer**  **diagnosis to treatment** | |  |  |  |  | 0.764 |  |  |  |  | 0.427 |
|  | ≤ 30 days | 12,492 | 94.03 | 1,243 | 93.53 |  | 8,792 | 82.85 | 863 | 81.26 |  |
|  | 31~150 days | 581 | 4.37 | 63 | 4.74 |  | 1,116 | 10.52 | 122 | 11.49 |  |
|  | ≥ 151 days | 212 | 1.60 | 23 | 1.73 |  | 704 | 6.63 | 77 | 7.25 |  |
| **Gender** | |  |  |  |  | 0.901 |  |  |  |  | 1.000 |
|  | Female | 5,727 | 43.11 | 570 | 42.89 |  | 4,491 | 42.32 | 450 | 42.37 |  |
|  | Male | 7,558 | 56.89 | 759 | 57.11 |  | 6,121 | 57.68 | 612 | 57.63 |  |
| **Age** | |  |  |  |  | 0.611 |  |  |  |  | 0.546 |
|  | ≤ 44 | 1,021 | 7.69 | 111 | 8.35 |  | 950 | 8.95 | 97 | 9.13 |  |
|  | 45~54 | 2,033 | 15.30 | 213 | 16.03 |  | 1,801 | 16.97 | 200 | 18.83 |  |
|  | 55~64 | 3,116 | 23.46 | 304 | 22.87 |  | 2,334 | 21.99 | 222 | 20.90 |  |
|  | 65~74 | 3,478 | 26.18 | 327 | 24.60 |  | 2,684 | 25.29 | 256 | 24.11 |  |
|  | ≥ 75 | 3,637 | 27.38 | 374 | 28.14 |  | 2,843 | 26.79 | 287 | 27.02 |  |
| **Mean age** | | 64.79 | 13.51 | 64.62 | 13.73 | 0.661 | 64.05 | 13.96 | 63.87 | 13.99 | 0.676 |
| **Monthly salary** | |  |  |  |  | 0.284 |  |  |  |  | 0.802 |
|  | Low-income | 104 | 0.78 | 52 | 3.91 |  | 119 | 1.12 | 57 | 5.37 |  |
|  | ≤ 17280 | 499 | 3.76 |  | 0.00 |  | 442 | 4.17 |  | 0.00 |  |
|  | 17281~22800 | 6,534 | 49.18 | 643 | 48.38 |  | 5,264 | 49.60 | 533 | 50.19 |  |
|  | 22801~28800 | 2,609 | 19.64 | 266 | 20.02 |  | 2,034 | 19.17 | 213 | 20.06 |  |
|  | 28801~36300 | 934 | 7.03 | 77 | 5.79 |  | 727 | 6.85 | 77 | 7.25 |  |
|  | 36301~45800 | 1,140 | 8.58 | 124 | 9.33 |  | 944 | 8.90 | 89 | 8.38 |  |
|  | ≥ 45801 | 1,465 | 11.03 | 167 | 12.57 |  | 1,082 | 10.20 | 93 | 8.76 |  |
| **Urbanization level** | |  |  |  |  | 0.480 |  |  |  |  | 0.708 |
|  | Level 1 | 3,781 | 28.46 | 389 | 29.27 |  | 3,155 | 29.73 | 318 | 29.94 |  |
|  | Level 2 | 3,885 | 29.24 | 354 | 26.64 |  | 3,100 | 29.21 | 304 | 28.63 |  |
|  | Level 3 | 2,103 | 15.83 | 229 | 17.23 |  | 1,612 | 15.19 | 146 | 13.75 |  |
|  | Level 4 | 1,933 | 14.55 | 204 | 15.35 |  | 1,492 | 14.06 | 160 | 15.07 |  |
|  | Level 5 | 416 | 3.13 | 42 | 3.16 |  | 288 | 2.71 | 36 | 3.39 |  |
|  | Level 6 | 536 | 4.03 | 49 | 3.69 |  | 479 | 4.51 | 49 | 4.61 |  |
|  | Level 7 | 631 | 4.75 | 62 | 4.67 |  | 486 | 4.58 | 49 | 4.61 |  |
| **CCI score** | |  |  |  |  | 0.047 |  |  |  |  | 0.697 |
|  | ≤ 3 | 9,372 | 70.55 | 913 | 68.70 |  | 4,049 | 38.15 | 418 | 39.36 |  |
|  | 4~6 | 1,888 | 14.21 | 222 | 16.70 |  | 2,145 | 20.21 | 215 | 20.24 |  |
|  | ≥ 7 | 2,025 | 15.24 | 194 | 14.60 |  | 4,418 | 41.63 | 429 | 40.40 |  |
| **Catastrophic illness** | |  |  |  |  | 0.155 |  |  |  |  | 0.069 |
|  | No | 12,883 | 96.97 | 1,298 | 97.67 |  | 10,182 | 95.95 | 1,006 | 94.73 |  |
|  | Yes | 402 | 3.03 | 31 | 2.33 |  | 430 | 4.05 | 56 | 5.27 |  |
| **Joint MDT care** | |  |  |  |  | 0.843 |  |  |  |  | 0.416 |
|  | No | 11,507 | 86.62 | 1,148 | 86.38 |  | 9,552 | 90.01 | 947 | 89.17 |  |
|  | Yes | 1,778 | 13.38 | 181 | 13.62 |  | 1,060 | 9.99 | 115 | 10.83 |  |
| **Hospital level** | |  |  |  |  | 0.162 |  |  |  |  | 0.929 |
|  | Major medical center | 8,945 | 67.33 | 933 | 70.20 |  | 6,880 | 64.83 | 682 | 64.22 |  |
|  | Regional hospital | 4,151 | 31.25 | 376 | 28.29 |  | 3,472 | 32.72 | 356 | 33.52 |  |
|  | District hospital | 153 | 1.15 | 20 | 1.50 |  | 234 | 2.21 | 24 | 2.26 |  |
|  | Others | 36 | 0.27 |  | 0.00 |  | 26 | 0.25 |  | 0.00 |  |
| **Hospital ownership** | |  |  |  |  | 0.973 |  |  |  |  | 0.482 |
|  | Public | 3,840 | 28.90 | 383 | 28.82 |  | 3,385 | 31.90 | 327 | 30.79 |  |
|  | Private | 9,445 | 71.10 | 946 | 71.18 |  | 7,227 | 68.10 | 735 | 69.21 |  |
| **Hospital service volume** | |  |  |  |  | 0.947 |  |  |  |  | 0.794 |
|  | Low | 2,911 | 21.91 | 286 | 21.52 |  | 2,865 | 27.00 | 297 | 27.97 |  |
|  | Middle | 6,768 | 50.94 | 680 | 51.17 |  | 5,167 | 48.69 | 511 | 48.12 |  |
|  | High | 3,606 | 27.14 | 363 | 27.31 |  | 2,580 | 24.31 | 254 | 23.92 |  |
